# Altered Expression of NF-***κ***B and SP1 after Exposure to Advanced Glycation End-Products and Effects of Neurotrophic Factors in AGEs Exposed Rat Retinas

**DOI:** 10.1155/2015/543818

**Published:** 2015-05-20

**Authors:** Guzel Bikbova, Toshiyuki Oshitari, Takayuki Baba, Shuichi Yamamoto

**Affiliations:** Department of Ophthalmology and Visual Science, Chiba University Graduate School of Medicine, Inohana 1-8-1, Chuo-ku, Chiba, Chiba Prefecture 260-8670, Japan

## Abstract

To determine the effect of advanced glycation end-products (AGEs) on neurite regeneration, and also to determine the regenerative effects of different neurotrophic factors (NTFs) on rat retinal explants, the retinas of SD rats were cultured in three-dimensional collagen gels and incubated in 6 types of media: (1) serum-free control culture media; (2) 100 *μ*g/mL AGEs-BSA media; (3) AGEs-BSA + 100 ng/mL neurotrophin-4 (NT-4) media; (4) AGEs-BSA + 100 ng/mL hepatocyte growth factor media; (5) AGEs-BSA + 100 ng/mL glial cell line-derived neurotrophic factor media; or (6) AGEs-BSA + 100 *µ*M tauroursodeoxycholic acid media. After 7 days, the number of regenerating neurites was counted. The explants were immunostained for nuclear factor-*κ*B (NF-*κ*B) and specificity protein 1 (SP1). Statistical analyses were performed by one-way ANOVA. In retinas incubated with AGEs, the numbers of neurites were fewer than in control. All of the NTFs increased the number of neurites, and the increase was more significant in the NT-4 group. The number of NF-*κ*B and SP1 immunopositive cells was higher in retinas exposed to AGEs than in control. All of the NTFs decreased the number of NF-*κ*B immunopositive cells but did not significantly affect SP1 expression. These results demonstrate the potential of the NTFs as axoprotectants in AGEs exposed retinal neurons.

## 1. Introduction

Advanced glycation end-products (AGEs) have been shown to accumulate in various tissues under diabetic conditions, and they participate in the development of vascular complications such as diabetic retinopathy [1, 2]. Our recent results showed that even a low concentration of AGEs, for example, 10 *μ*g/mL, can induce neuronal apoptosis in retinal neurons and decrease the number of regenerating neurites in cultures [3].

AGEs accomplish their effects by binding to specific cellular receptors [4, 5], such as receptors for AGEs (RAGEs). These receptors have been found on neurons, mesangial cells, smooth muscle cells, and endothelial cells [6–8]. The binding of RAGE to AGEs precursors generates intracellular oxidative stress which then induces receptor-mediated production of reactive oxygen species. This then results in the activation of the free radical-sensitive transcription factor nuclear factor-*κ*B (NF-*κ*B) [9, 10]. The activated NF-*κ*B translocates into the nucleus and causes pathological changes in gene expression [9, 11–13].

Specificity protein 1 (SP1) is a transcription factor that either activates or represses transcription in response to physiological and pathological stimuli. It regulates the expression of a large number of genes involved in a variety of processes such as cell growth, apoptosis, differentiation, and immune responses [14]. Several genes can be regulated by a combination of NF-*κ*B and SP1, and in specific cases by direct interaction between the NF-*κ*B protein and SP1 protein [15].

Tanaka et al. [16] found that AGEs can activate the* RAGE* gene through NF-*κ*B and SP1, causing enhanced AGE-RAGE interactions in human vascular endothelial cells. They concluded that this activation can exacerbate diabetic microvasculopathy [16]. However, there are no reports demonstrating the relationship between NF-*κ*B/SP1 expression and regeneration in AGE exposed retinal neurons.

Growing evidence indicates that neuronal abnormalities such as neuronal cell death and vascular abnormalities are associated with the development of early diabetic retinopathy [17]. However, the precise mechanism causing neuronal cell death remains undetermined. Because neuronal cell death is an irreversible change and can affect visual function of diabetic eyes, neuroprotective and regenerative therapies need to be determined [17]. However, no reports have been simultaneously compared the neuroprotective and regenerative effects of several neurotrophic factors including neurotrophin-4 (NT-4), hepatocyte growth factor (HGF), glial cell line-derived neurotrophic factor (GDNF), and tauroursodeoxycholic acid (TUDCA) in AGE exposed retinas cultured in the same system.

The purpose of this study was to examine the effect of high doses of AGEs on neuronal cell death and neurite regeneration in isolated rat retinas. We also determined the neuroprotective and regenerative effects of four neurotrophic factors, namely, NT-4, HGF, GDNF, and TUDAC in AGE exposed retinas. In addition, we also examined whether the expressions of NF-*κ*B and SP1 were correlated with the neuroprotective and regenerative effects of different neurotrophic factors in AGEs exposed rat retinas.

## 2. Materials and Methods

### 2.1. Animals

Seven-week-old male Sprague-Dawley (SD) rats (Japan SLC Co., Hamamatsu, Japan) were used. All of the procedures were performed in accordance with the ARVO Statement for the Use of Animals in Ophthalmic and Vision Research.

### 2.2. Three-Dimensional Collagen Gel Culture of Rat Retinal Explants

Six SD rats were killed by an overdose of ether. The retinas were isolated under sterile conditions and cut into square pieces of 0.16 mm^2^ with sharp razor blades. Then the retinal explants were cultured on three-dimensional collagen gels as described in detail [18–23]. The retinal explants were incubated in 6 different types of media; (1) serum-free control culture media, (2) 100 *μ*g/mL glucose-AGE-BSA (Cyclex Co., Nagano, Japan) or glycolaldehyde-AGE-BSA (Cyclex Co) or glyceraldehyde-AGE-BSA (Cyclex Co) media, (3) glucose-AGE or glycolaldehyde-AGE or glyceraldehyde-AGE + 100 ng/mL NT-4 (R&D Systems, Minneapolis, MN) media, (4) glucose-AGE, glycolaldehyde-AGE, or glyceraldehyde-AGE + 100 ng/mL HGF (R&D systems) media, (5) glucose-AGE, or glycolaldehyde-AGE, or glyceraldehyde-AGE + 100 ng/mL GDNF (R&D Systems) media, and (6) glucose-AGE, or glycolaldehyde-AGE, or glyceraldehyde-AGE + 100 *μ*M TUDCA (WAKO, Osaka, Japan) media.

The explants were maintained at 37°C and exposed to 5% CO_2_. The serum-free media contained 7.5 mM glucose, 5 *μ*g/mL insulin, 16.1 *μ*g/mL putrescine, 10% bovine serum albumin, 3.7 mg/mL NaHCO_3_, 5.2 mg/L Na_2_SeO_3_, and 3.6 mg/mL HEPES in minimum essential medium as described [21, 23, 24]. One hundred *μ*g/mL BSA was added to the control medium (N + A) as a control for the concentration of AGE-BSA.

### 2.3. TUNEL Staining

To determine whether apoptosis had occurred, the retinal explants were fixed in 4% paraformaldehyde after 7 days in culture and sectioned on a cryostat. Then, TdT-dUTP terminal nick-end labeling (TUNEL) staining was carried out with an apoptosis detection kit (Chemicon International, Temecula, CA) according to the manufacturer's instructions. Nonspecific signals were detected by omitting the enzyme reaction. Sections were costained with 4,6-diamidino-2-phenyl indole (DAPI, Polyscience Inc., Warrington, PA). For quantitative analyses, the ratio of the number of TUNEL-positive cells to the total number of DAPI-staining nuclei in the ganglion cell layer (GCL) was determined. A total of 18 sections from the 6 explants/group were studied, and the results were used for the statistical analyses. The total number of nuclei counted was 405 (N), 215 (N + A), 816 (AGEs), 472 (NT-4), 396 (HGF), 512 (GDNF), and 494 (TUDCA).

### 2.4. Immunohistochemistry

The retinal explants were fixed as described and cryosections were cut. After blocking the sections in 5% goat serum and 3% bovine serum in 0.1 M phosphate buffer saline, they were incubated with antibodies against rabbit anti-phosphorylated NF-*κ*B (p-NF-*κ*B) and SP1 transcription factor (Santa Cruz Biotechnology, Santa Cruz, CA) at 4°C overnight. Then, the sections were incubated with fluorescein isothiocyanate-conjugated anti-rabbit IgG for one hour. Sections were costained with DAPI to make the nuclei visible. The number of p-NF-*κ*B- and SP1-positive cells in the GCL was counted. For quantitative analyses, the number of immunopositive cells in the GCL was expressed relative to the total number of DAPI-stained nuclei. Eighteen sections were used from the 6 explants/group. The total number of nuclei counted was 301 in N, 234 in AGEs, 198 in NT-4, 254 in HGF, 186 in GDNF, and 276 in TUDCA.

### 2.5. Assessment of Regenerating Neurites

The number of neurites regenerated from the explants was counted under a phase-contrast microscope after 7 days in culture when the number of regenerating neurites was very high [3, 18, 21, 23, 24]. Branched neurites were counted as one. The number of explants examined was 101 in the control group including that in serum-free media (N, N + A, N + NT-4, N + HGF, N + GDNF, N + TUDCA), 11 in the glucose-AGE-BSA group, 19 in the glycolaldehyde-AGE-BSA group, 18 in the glyceraldehyde-AGE-BSA group, 14 in the glucose-AGE-BSA + NT-4, 12 in the glycolaldehyde-AGE-BSA + NT-4, and 11 in the glyceraldehyde-AGE-BSA + NT-4 groups, 12 in the glucose-AGE-BSA + HGF, 14 in the glycolaldehyde-AGE-BSA + HGF, 13 in the glyceraldehyde-AGE-BSA + HGF groups, 12 in the glucose-AGE-BSA + GDNF, 13 in the glycolaldehyde-AGE-BSA + GDNF, 14 in the glyceraldehyde-AGE-BSA + GDNF groups, 10 in the glucose-AGE-BSA + TUDCA, 11 in the glycolaldehyde-AGE-BSA + TUDCA, and 10 in the glyceraldehyde-AGE-BSA + TUDCA groups.

Statistical analyses were carried by one-way ANOVA with Scheffe's *F* tests. A *P* < 0.05 was considered significant.

## 3. Results

### 3.1. Detection of Apoptosis

**Figure 1:**
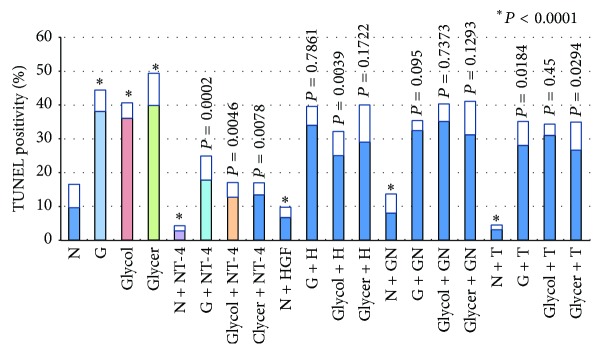
Graph showing the ratio of TUNEL-positive cells to all cells in the GCL of retinal explants. N, serum-free media; G, glucose-AGE-BSA; Glycol, glycolaldehyde-AGE-BSA; Glycer, glyceraldehyde-AGE-BSA; NT-4, neurotrohin-4; H, hepatocyte growth factor; GN, glial cell line-derived neurotrophic factor; T, tauroursodeoxycholic acid.

To determine whether AGEs were toxic to the retinas in culture, the number of TUNEL-positive cells in the GCL was counted. The majority of the TUNEL-positive cells were detected in the GCL because all of the retinal ganglion cells (RGCs) were axotomized to isolate the retina [3, 18, 21, 24–26]. In retinas added 100 *μ*g/mL BSA more to the control medium (N + A), the number of TUNEL-positive cells was not significantly different from the control medium (N) (9.3 ± 2.3% versus 9.7 ± 6.8%, *P* = 0.244). In retinas cultured in glucose-AGE-BSA, glycolaldehyde-AGE-BSA, and glyceraldehyde-AGE-BSA, the number of TUNEL-positive cells in the GCL was significantly higher than that in the serum-free control medium (38.1 ± 6.2% versus 9.7 ± 6.8%, *P* < 0.0001; 36.1 ± 4.9% versus 9.7 ± 6.8%, *P* < 0.0001; and 39.9 ± 9.4% versus 9.7 ± 6.8%, *P* < 0.0001, resp.; Figure 1). The addition of NT-4 decreased the number of TUNEL-positives cells more than in glucose-AGE-BSA without NT-4 (17.8 ± 7.2% versus 38.1 ± 6.2%; *P* = 0.0002), in glycolaldehyde-AGE-BSA without NT-4 (12.8 ± 4.2% versus 36.1 ± 4.0%; *P* = 0.0046), and in glyceraldehyde-AGE-BSA without NT-4 (13.4 ± 3.6% versus 39.9 ± 9.4%; *P* = 0.0078; Figure 1).

Addition of HGF in AGE-BSA did not decrease the number of TUNEL-positives cells compared to the number in glucose-AGE-BSA without HGF (34.0 ± 5.5% versus 38.1 ± 6.2%; *P* = 0.7861) and glyceraldehyde-AGE-BSA without HGF (29.1 ± 11.0% versus 39.9 ± 9.4%; *P* = 0.1722) but it significantly decreased in glycolaldehyde-AGE-BSA without HGF (25.4% ± 7.3% versus 36.1 ± 4.0%; *P* = 0.0039; Figure 1).

Addition of GDNF did not decrease the number of TUNEL-positives cells compared to the number in glucose-AGE-BSA without GDNF (32.4 ± 3.0% versus 38.1 ± 6.2%; *P* = 0.0935) and in glycolaldehyde-AGE-BSA without GDNF (35.2% ± 5.1% versus 36.1 ± 4.0%; *P* = 0.7373) and in glyceraldehyde-AGE-BSA without GDNF (31.2 ± 9.78% versus 39.9 ± 9.4%; *P* = 0.1293; Figure 1).

The addition of TUDCA decreased the number of TUNEL-positives cells more than that in glucose-AGE-BSA without TUDCA (28.2 ± 6.5% versus 38.1 ± 6.2%; *P* = 0.0184) and in glyceraldehyde-AGE-BSA without TUDCA (26.6 ± 8.3% versus 39.9 ± 9.4%; *P* = 0.0294) but it did not decrease in glycolaldehyde-AGE-BSA without TUDCA (31.6 ± 3.3% versus 36.1 ± 4.0%; *P* = 0.45; Figure 1).

### 3.2. p-NF-*κ*B Immunopositivity in Ganglion Cell Layer

**Figure 2:**
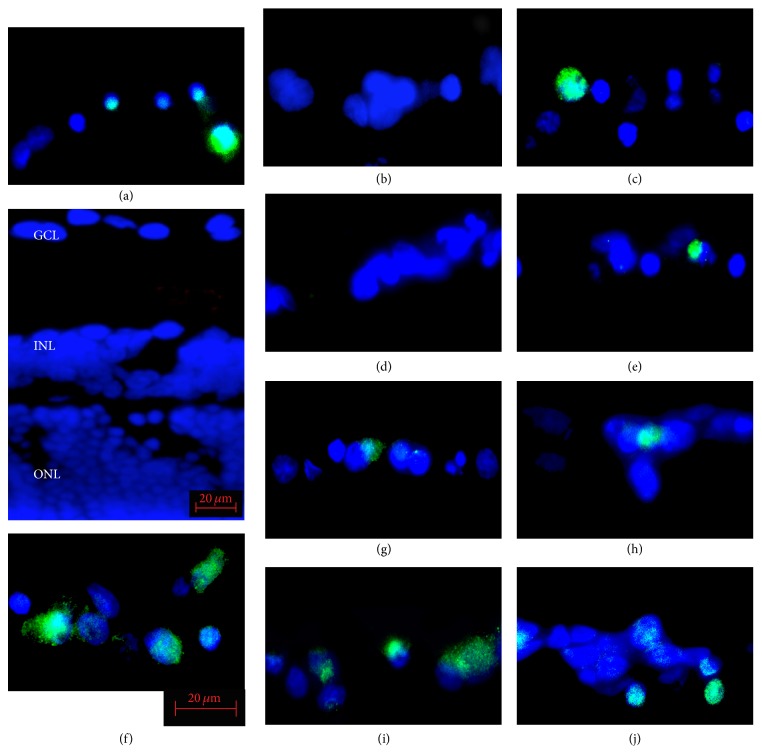
Representative photomicrographs of NF-*κ*B immunopositive cells in the ganglion cell layer (GCL) of isolated rat retinas. In retinas cultured in glucose-AGE-BSA (f), the number of immunopositive cells in the GCL is significantly higher than in serum-free control media (a). In AGEs exposed retinas supplemented with NT-4 (glucose-AGE-BSA + NT-4) (g), with HGF (glucose-AGE-BSA + HGF) (h), with GDNF (glucose-AGE-BSA + GDNF) (i), and with TUDCA (glucose-AGE-BSA + TUDCA) (j) the number of NF-*κ*B immunopositive cells is fewer than that in AGEs exposed retinas without neurotrophic factors. The blue staining shows the DAPI-stained nuclei. Panels (b), (c), (d), and (e) are pictures of the serum-free media supplemented with NT-4 (b), HGF (c), GDNF (d), and TUDCA (e). INL, inner nuclear layer; ONL, outer nuclear layer. Bar = 20 *μ*m.

**Figure 3:**
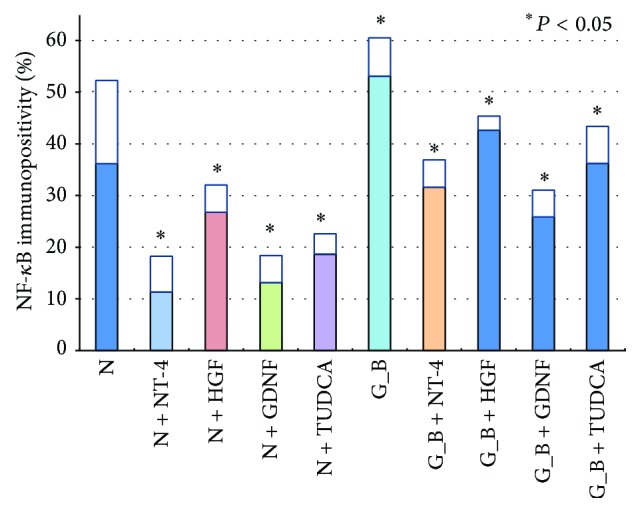
Graph showing the ratio of NF-*κ*B immunopositive cells to all cells in the GCL of retinal explants. N, serum-free media; G_B, glucose-AGE-BSA; Glycol, glycolaldehyde-AGE-BSA; Glycer, glyceraldehyde-AGE-BSA; NT-4, neurotrohin-4; HGF, hepatocyte growth factor; GDNF, glial cell line-derived neurotrophic factor; TUDCA, tauroursodeoxycholic acid.

The sections were immunostained for p-NF-*κ*B to determine whether NF-*κ*B was activated in retinas exposed to AGEs. The effects of the neurotrophic factors on the activation of NF-*κ*B were also examined. In retinas cultured with glucose-AGE-BSA, the number of p-NF-*κ*B immunopositive cells was higher than in serum-free control medium (53.2 ± 7.2% versus 31.6 ± 16.1%; *P* = 0.0146; Figures 2 and 3). Addition of NT-4 decreased the number of immunopositive cells more than in the serum-free media (11.3 ± 6.9% versus 31.6 ± 16.1%; *P* = 0.0315) and more than in glucose-AGE-BSA without NT-4 (31.5 ± 5.3% versus 53.2 ± 7.2%; *P* = 0.0133; Figures 2 and 3). Addition of HGF decreased the number of immunopositive cells more than in serum-free media (26.8 ± 5.3% versus 31.6 ± 16.1%; *P* = 0.049) and in glucose-AGE-BSA without HGF (42.6 ± 2.7% versus 53.2 ± 7.2%; *P* = 0.0114; Figures 2 and 3). Addition of GDNF decreased the number of immunopositive cells more than in serum-free media (13.3 ± 5.0% versus 31.6 ± 16.1%; *P* = 0.0495) and in glucose-AGE-BSA without GDNF (25.8 ± 5.3% versus 53.2 ± 7.2%; *P* = 0.0011; Figures 2 and 3). Addition of TUDCA decreased the number of immunopositive cells more than in serum-free media (18.6 ± 3.97% versus 31.6 ± 16.1%; *P* = 0.0024) and in glucose-AGE-BSA without TUDCA (36.2 ± 7.2% versus 53.2 ± 7.2%; *P* = 0.0034; Figures 2 and 3).

### 3.3. SP1 Immunopositivity in Ganglion Cell Layer

**Figure 4:**
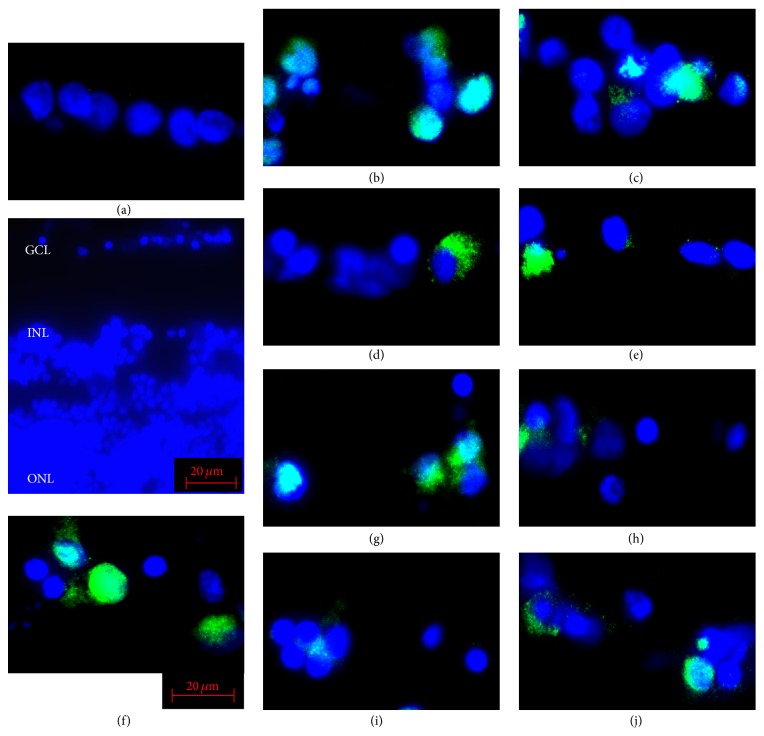
Representative photomicrographs of SP1 immunopositive cells in the ganglion cell layer (GCL) of isolated rat retinas. In retinas cultured in glucose-AGE-BSA (f), the number of immunopositive cells in the GCL is significantly higher than in serum-free control media (a). In AGEs exposed retinas supplemented with NT-4 (glucose-AGE-BSA + NT-4) (g), with HGF (glucose-AGE-BSA + HGF) (h), with GDNF (glucose-AGE-BSA + GDNF) (i), and with TUDCA (glucose-AGE-BSA + TUDCA) (j) the number of SP1 immunopositive cells is higher than that in AGEs exposed retinas without neurotrophic factors. The blue signals show the DAPI-stained nuclei. Panels (b), (c), (d), and (e) show the representative pictures of the serum-free media incubated with NT-4 (b), HGF (c), GDNF (d), and TUDCA (e). INL, inner nuclear layer; ONL, outer nuclear layer. Bar = 20 *μ*m.

**Figure 5:**
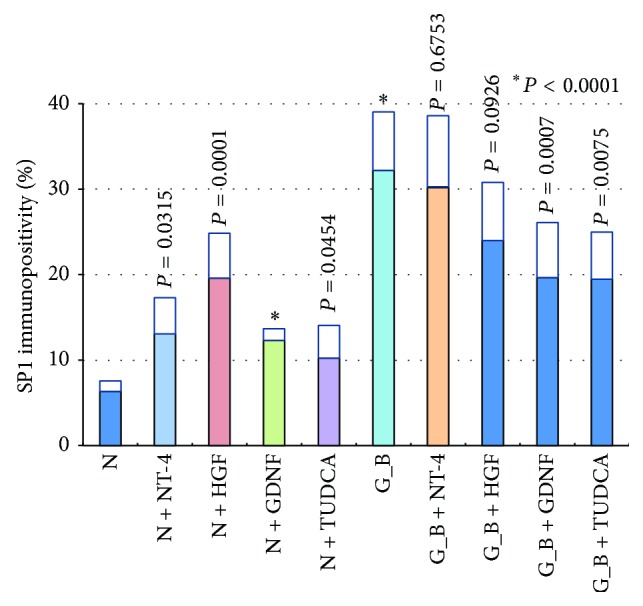
Graph showing the ratio of SP1 immunopositive cells to all cells in the GCL of retinal explants. N, serum free media, G_B, glucose-AGE-BSA; Glycol, glycolaldehyde-AGE-BSA; Glycer, glyceraldehyde-AGE-BSA; NT-4, neurotrohin-4; HGF, hepatocyte growth factor, GDNF, glial cell line-derived neurotrophic factor; TUDCA, tauroursodeoxycholic acid.

The sections were immunostained for SP1 transcription factor to determine whether it was expressed in retinas exposed to AGEs. We also examined the effect of neurotrophic factors on the expression of SP1 transcription factor. In retinas cultured with glucose-AGE-BSA, the number of SP1 immunopositive cells was higher than in serum-free control medium (32.2 ± 6.8% versus 6.3 ± 1.2%; *P* < 0.0001; Figures 4 and 5). Addition of NT-4 increased the number of immunopositive cells more than in serum-free media (13.0 ± 4.3% versus 6.3 ± 1.2%; *P* = 0.0315) but did not decrease the number of immunopositive cells in glucose-AGE-BSA without NT-4 (30.2 ± 8.4% versus 32.2 ± 6.8%; *P* = 0.6753; Figures 4 and 5). Addition of HGF increased the number of immunopositive cells more than in serum-free media (19.7 ± 5.16% versus 6.3 ± 1.2%; *P* = 0.0001) but did not decrease the number of immunopositive cells in glucose-AGE-BSA without HGF (24.0 ± 6.7% versus 32.2 ± 6.8%; *P* = 0.0926; Figures 4 and 5). Addition of GDNF increased the number of immunopositive cells more than in the serum-free media (12.3 ± 1.4% versus 6.3 ± 1.2%; *P* < 0.0001) but it decreased compared to in glucose-AGE-BSA without GDNF (19.7 ± 6.4% versus 32.2 ± 6.8%; *P* = 0.0007; Figures 4 and 5). Addition of TUDCA increased the number of immunopositive cells more than in serum-free media (10.2 ± 3.9% versus 6.3 ± 1.2%; *P* = 0.0454) but it decreased compared to in glucose-AGE-BSA without TUDCA (19.5 ± 5.5% versus 32.2 ± 6.8%; *P* = 0.0075; Figures 4 and 5).

### 3.4. Regenerating Neurites

**Figure 6:**
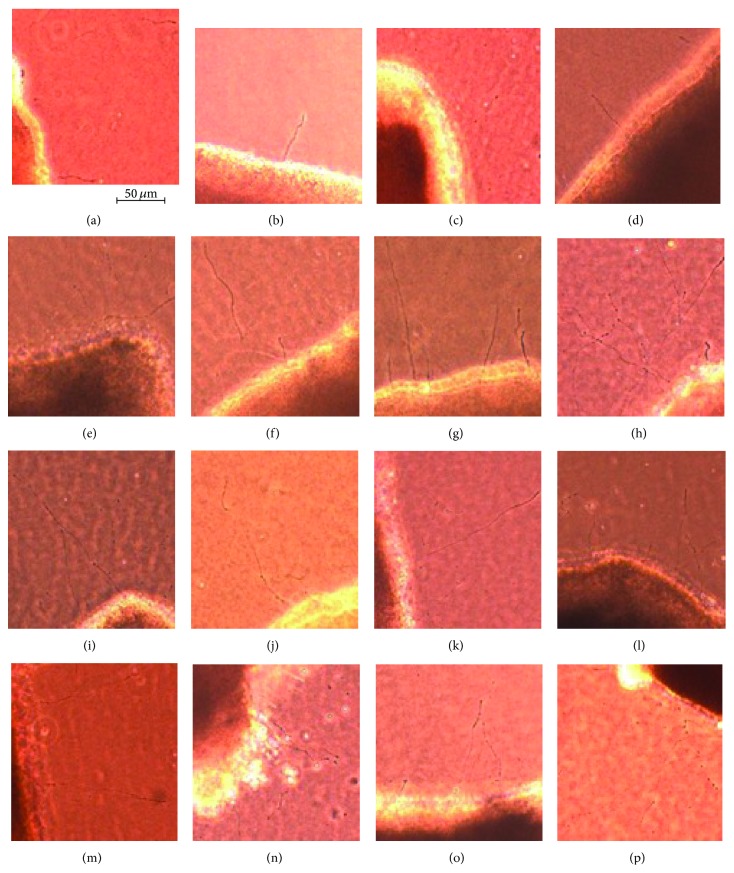
Representative photographs of regenerating neurites. Regenerating neurites are seen under phase-contrast microscopy. In the control serum-free media (a) neurites with normal length are present. In retinas cultured in glucose-AGE-BSA (b), glyceraldehyde-AGE-BSA (c), and glycolaldehyde-AGE-BSA (d), the neurites were shorter, and the numbers of neurites were fewer. In AGEs exposed retinas supplemented with NT-4 (glucose-AGE-BSA + NT-4 (e), glyceraldehyde-AGE-BSA + NT-4 (f), and glycolaldehyde-AGE-BSA + NT-4 (g)), the neurites are longer and thicker, and the number of neurites are higher even than in serum-free control media (a). In AGEs exposed retinas supplemented with HGF, GDNF, and TUDCA (glucose-AGE-BSA + HGF (h), glyceraldehyde-AGE-BSA + HGF (i), and glycolaldehyde-AGE-BSA + HGF (j)), (glucose-AGE-BSA + GDNF (k), glyceraldehyde-AGE-BSA + GDNF (l), and glycolaldehyde-AGE-BSA + GDNF (m)), (glucose-AGE-BSA + TUDCA (n), glyceraldehyde-AGE-BSA + TUDCA (o), and glycolaldehyde-AGE-BSA + TUDCA (p)), the neurites are longer and thicker than in AGEs exposed retinas.

**Figure 7:**
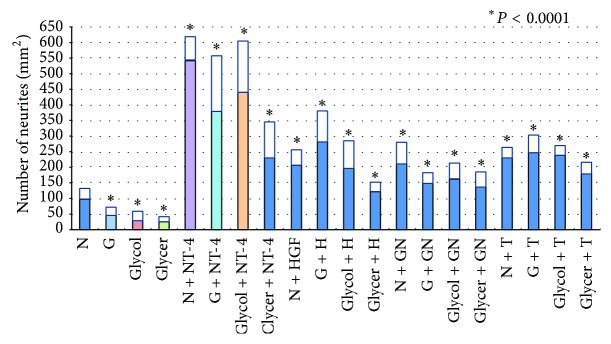
Graph showing the number of regenerating neurites in all groups. N, serum-free media, G, glucose-AGE-BSA; Glycol, glycolaldehyde-AGE-BSA; Glycer, glyceraldehyde-AGE-BSA; NT-4, neurotrohin-4; H, hepatocyte growth factor; GN, glial cell line-derived neurotrophic factor; T, tauroursodeoxycholic acid.

In retinas added 100 *μ*g/mL BSA more to the control medium (N + A), the number of neurites was not significantly different from the control medium (N) (94.6 ± 24.0/mm^2^ versus 97.5 ± 34.9/mm^2^, *P* = 0.948). In retinas incubated with AGEs (glucose-AGE, glycolaldehyde-AGE, and glyceraldehyde-AGE), the number of regenerating neurites was less than in retinas without AGE (45.0 ± 27.5/mm^2^ versus 97.5 ± 34.9/mm^2^, *P* = 0.0046; 29.4 ± 29.4/mm^2^ versus 97.5 ± 34.9/mm^2^, *P* = 0.0003; 25.0 ± 15.6/mm^2^ versus 97.5 ± 34.9/mm^2^, *P* < 0.0001; Figures 6 and 7). All of the retinas incubated in the neurotrophic factors (NT-4, HGF, GDNF, and TUDCA) had an increase in the number of regenerating neurites in serum-free media (541.9 ± 77.5/mm^2^ versus 97.5 ± 34.9/mm^2^, *P* < 0.0001; 207.5 ± 49.1/mm^2^ versus 97.5 ± 34.9/mm^2^, *P* < 0.0001; 211.3 ± 70.6/mm^2^ versus 97.5 ± 34.9/mm^2^, *P* < 0.0001; 229.4 ± 33.8/mm^2^ versus 97.5 ± 34.9/mm^2^, *P* < 0.0001; Figures 6 and 7). In addition, all of the neurotrophic factors increased the number of regenerated neurites in AGEs exposed retinas, but the most significant regenerative effect was found in the NT-4 group: 379.4 ± 178.0/mm^2^ versus 45.0 ± 27.5/mm^2^, *P* < 0.0001, in NT-4 group; 281.3 ± 100.6/mm^2^ versus 45.0 ± 27.5/mm^2^, *P* < 0.0001, in HGF group; 148.8 ± 35.0/mm^2^ versus 45.0 ± 27.5/mm^2^, *P* < 0.0001, in the GDNF group; 247.5 ± 56.9/mm^2^ versus 45.0 ± 27.5/mm^2^, *P* < 0.0001, in TUDCA group supplemented with glucose-AGE incubated retinas; 440.0 ± 165.0/mm^2^ versus 29.4 ± 29.4/mm^2^, *P* < 0.0001, in NT-4 group; 196.3 ± 89.4/mm^2^ versus 29.4 ± 29.4/mm^2^, *P* < 0.0001, in HGF group; 161.1 ± 53.9/mm^2^ versus 29.4 ± 29.4/mm^2^, *P* < 0.0001, in GDNF group; 238.1 ± 33.1/mm^2^ versus 29.4 ± 29.4/mm^2^, *P* < 0.0001, in TUDCA group supplemented to glycolaldehyde-AGE incubated retinas, 230.6 ± 116.9/mm^2^ versus 25.0 ± 15.6/mm^2^, *P* < 0.0001, in NT-4 group; 123.8 ± 29.4/mm^2^ versus 25.0 ± 15.6/mm^2^, *P* < 0.0001, in HGF group; 137.5 ± 48.8/mm^2^ versus 25.0 ± 15.6/mm^2^, *P* < 0.0001, in GDNF group; 178.8 ± 39.4/mm^2^ versus 25.0 ± 15.6/mm^2^, *P* < 0.0001, in TUDCA group supplemented to glyceraldehyde-AGE incubated retinas (Figures 6 and 7).

## 4. Discussion

The importance of AGEs in the pathogenesis of diabetic complications has been shown in animal models. Two unrelated AGE inhibitors were found to partially block some functional and structural changes in retinas, neuronal tissues, and kidneys of diabetic animal models [27–29]. Our previous study showed that even a low concentration of AGEs, for example, 10 *μ*g/mL, induced neuronal apoptosis in the GCL and decreased the number of regenerated neurites in culture. Higher doses (100 *μ*g/mL) of AGEs had similar effects as low concentrations and decreased the number of TUNEL-positive cells and significantly blocked neurite regeneration [3].

The results of this study confirmed that the apoptosis in the GCL were caused by AGEs and also that neurite regeneration was significantly suppressed. All examined neurotrophic factors were able to increase the number of regenerative neurites; however the most significant regenerative effect was observed with NT-4. In addition, all examined neurotrophic factors suppressed the expression of NF-*κ*B expression in AGEs exposed retinas.

NF-*κ*B is a primary transcription factor that plays an important role in regulating cellular responses, that is, a transcription factor that is present in cells in an inactive state and does not require new protein synthesis to become activated. Other members of this family include transcription factors such as c-Jun, STATs, and nuclear hormone receptors. This allows NF-*κ*B to be the first responder to toxic cellular stimuli. Some aspects of the mechanism by which NF-*κ*B protects cells against toxins have been identified. For example, tumor necrosis factor-*α* has been shown to protect hippocampal neurons against excitatory amino acid toxicity through NF-*κ*B activation by inducing Bcl-2 and Bcl-x expression [30].

SP1 transcription factor belongs to a group of factors that are associated with GC-rich promoters that are involved in basal promoter activity. SP1 regulates the expression of different genes, including the vascular endothelial growth factor, fibrogenic cytokine, and many matrix genes [31]. Several studies reported that the interactions between AGE and RAGE cause phenotypic changes in the microvascular endothelial cells and pericytes [32–38]. It was shown that upregulations of RAGEs are mediated by NF-*κ*B and SP1 [16].

Our results showed that AGEs increase the expression of the transcription factor NF-*κ*B and SP1 in retinal neurons. This suggests that AGEs enhanced the expression of the RAGEs gene in retinal neurons through the increased expression of NF-*κ*B and SP1. An upregulation of NF-*κ*B and SP1 proteins in retinal neurons suggests that different protective mechanisms are important for the protection of these cells against AGEs-mediated cell death.

TUDCA is a member of a group of compounds that modulate the endoplasmic reticulum (ER) function, protecting the cells against ER stress-induced apoptosis [39]. Earlier studies showed that TUDCA had protective effects on damaged retinal neurons under diabetic stress as an anti-ER reagent. The neuroprotective effect of TUDCA was correlated with the suppression of phosphorylated JNK and phosphorylated c-Jun expression [18, 26].

HGF is a strong survival factor for developing and injured adult hepatocytes. It is also a potent mitogen and differentiation factor for endothelial cells [40], and it had neurotrophic and neuroprotective activity for central nervous system neurons [41, 42]. Tönges et al. [43] used an optic nerve axotomy model and demonstrated that HGF prevented RGC apoptosis in vivo in a concentration-dependent manner. This validated the beneficial role of HGF for retinal neurons [43]. Also Wong et al. [44] found that HGF promoted long-term survival and axonal regeneration of RGC after optic nerve injury [44].

Recently, GDNF was found to be a growth and survival factor for neuronal cells, and it promoted the survival of peripheral sensory and sympathetic neurons and also motor neurons [45]. Koeberle and Ball [46] studied the effects of GDNF on RGC survival and apoptosis after optic nerve transection. They suggested that GDNF aided in the survival of RGCs after axotomy [46].

NT-4 is a member of the nerve growth factor family which acts on different types of nerve cells, for example, sensory, cortical, and hippocampal neurons. It also acts on basal forebrain cholinergic nerve cells [47]. In an earlier study, we investigated the neuroprotective and regenerative effects of NT-4 on retinal neurons under diabetic condition [3, 18, 21, 26]. Our results showed that NT-4 promoted the survival and the regeneration of neuronal cells in the retinas incubated in high glucose media. The neuroprotective and regenerative effects of NT-4 were correlated with the reduction in the activation of caspase-9 and -3 [21], expression of PERK and CHOP [26], and c-Jun and JNK expression [18].

The results of the present study showed that all examined neurotrophic factors decreased the number of NF-*κ*B immunopositive cells in glucose-AGE-BSA exposed retina, but NT-4 had the highest significant effect. However, the number of SP1 immunopositive cells was increased by the addition of neurotrophic factors in serum-free media and more significantly in the HGF and NT-4 groups. Thus after the addition of neurotrophic factors to glucose-AGE-BSA media, the level of SP1 transcription factor remained high in the NT-4 and HGF group. However, there was a slight decrease in the GNDF and TUDCA groups.

Kanda et al. found that prostaglandin E2 promoted innervation in skin lesions by the induction of NT-4, and the induction was mediated by SP1 [48]. Their results showed that the EP3, G-protein-coupled receptors, mediated by prostaglandin E2 transcription of NT-4 was dependent on the activity of SP1. Thus, our findings suggest that SP1 may be related to neuronal survival and regeneration. Human NT-4 promoter has not been completely characterized. However, it may have several SP1-binding sites because antisense SP1 suppressed NT-4 expression [49].

It is possible that the increased expression of SP1 may result from an enhanced phosphorylation of SP1, because the SP1 promoter contains several SP1-binding elements and is positively regulated by its own gene product, SP1 protein [50]. Further studies are needed to identify the connection between SP1 expression and NT-4 transcription.

To find a possible decrease in the effect of the AGEs on the retina is important. Our findings that a suppression of NF-*κ*B expression in retinal neurons by several neurotrophic factors can result in neuroprotection, and reducing inflammation and oxidative stress suggests therapeutic potential of neuroprotective therapy in various ocular pathologies associated with AGEs accumulation.

In conclusion, high-dose AGEs inhibit neurite regeneration which is correlated with increased expression of NF-*κ*B and SP1. NT-4 enhances neurite regeneration in AGEs exposed retinas more than other neurotrophic factors such as HGF, GDNF, and TUDCA. This effect of NT-4 is correlated with NF-*κ*B suppression. SP1 overexpression may be related to neuronal regeneration in neurotrophic factors incubated retinas. Our results indicate the therapeutic potentials of the neurotrophic factors as axoprotectants in AGEs exposed retinas.
